# Cerebral and Retinal Neurovascular Changes: A Biomarker for Alzheimer’s Disease

**DOI:** 10.4172/2167-7182.1000447

**Published:** 2017-09-08

**Authors:** Karru Venkata Ravi Teja, TJM Tos Berendschot, Harry Steinbusch, AB Carroll Webers, R Praveen Murthy, PS Mathuranath

**Affiliations:** 1 Department of Neurology, National Institute of Mental Health and Neurosciences (NIMHANS), Bengaluru-560029, India; 2 Department of Neuroscience, National Institute of Mental Health and Neurosciences (NIMHANS), Bengaluru-560029, India; 3 Department of Translational Neuroscience, Faculty of Health, Medicine & Life Sciences, Maastricht University Medical Center, Universiteitssingel 40, Maastricht, The Netherlands; 4 University Eye Clinic Maastricht University Medical Center, Universiteitssingel 40, Maastricht, The Netherlands; 5 Prabha Eye Clinic, Bengaluru, India

**Keywords:** Alzheimer’s disease, Retina, Neurovascular unit, Biomarker

## Abstract

**Objectives:**

Biomarker quest for Alzheimer’s disease (AD) has gone a long way by studying various anatomical, physiological and biochemical parameters for detecting disease onset and predicting prognosis. Almost all the studies converge on the single hypothesis of the amyloid and Tau pathway. Recently, vascular hypothesis has evolved drawing attention towards a complex dynamic anatomical and physiological entity, neuro-vascular (NV) unit. Pathological changes at this level, altering the normal physiology such as auto-regulation and dynamics of blood brain barrier have been hypothesized as a probable basis for AD. This paper attempts to review the existing data on the vascular hypothesis and the current trends in analyzing the NV unit in AD.

**Design:**

This review initially focuses on the cerebral NV coupling followed by the retinal neurovascular coupling that mirrors the cerebral pathophysiology. The pathophysiology and the potential tools to diagnose AD at the level of NV unit are analyzed. Further, it examines the drawbacks in existing methods for analyzing the same.

**Findings:**

None of the current studies have emphasized the importance of studying the complex dynamic NV unit as a whole. This review strongly recommends the combination of vascular and neuro-glial parameters using objective methods for estimating the physiological and pathological changes in the NV unit.

**Discussion and conclusion:**

This review highlights the importance of retina for non-invasive estimation of the same. Also, novel algorithms for retinal image analysis have been proposed. The purpose of this review is to highlight the importance of retinal findings in neurodegenerative disorders and to create awareness among the neuroophthalmologists, of the potential benefits of ophthalmological tools in screening dementia patients.

## Introduction

The pathobiology underlying AD has been understood in a reasonable number of ways. Even after a century of its known existence, the amyloid-tau hypothesis takes the lead [1–2]. The pathobiology of AD as proposed by Braak [3] starts in the early childhood, progresses throughout the lifespan of the individual and culminates in end stage disease. This has raised the curiosity of the scientific world and generated various hypotheses. Next to amyloidogenic pathway is the vascular hypothesis where the neurovascular unit plays the central role.

## Methodology

In this review, the PUBMED search engine was used.

Period of review: 1976 to 2016.

Search terms used:

1. Optical coherence tomography and Alzheimer’s disease
2. Retina and dementia.
3. Neurovascular coupling and Alzheimer’s disease.

Both human as well as animal model studies are included. A total of 626 results were found and relevant articles highlighting the role of neurovascular coupling were selected. Recently two review articles were published on similar lines [4,5]. However, they fell short of analyzing the similarities and difficulties in analyzing the cerebral and retinal neurovascular units, which prompted us for this comprehensive review.

### Neurovascular unit

NV unit is an anatomical structure complex comprising of the neuron, glia and the blood vessel which auto-regulates blood flow through the NV coupling phenomenon. Blood supply to the brain is majorly through large intracranial vessels, internal carotid arteries, middle cerebral arteries, anterior cerebral arteries, posterior cerebral arteries, vertebral arteries and the basilar artery. These large arteries branch out to wrap the brain surface under the pia mater, thus called pial vessels; which in turn give rise to arterioles called parenchymal vessels that penetrate the brain parenchyma. These different blood vessels are regulated by different mechanisms. Large intracranial vessels are regulated by the autonomic nervous system [6,7], whereas the pial and the parenchymal vessels are under complex regulatory mechanisms [8–11]. In specific, parenchymal blood flow is auto-regulated at the NV unit [12–14]. Several studies have explored the physiology of NV unit, involving the anatomical structures, the synapses and the neurotransmitters released. In principal, synaptic activity increases the level of neurotransmitter specifically glutamate, which is taken by the NMDA receptors on the neuron and mGlu receptors on the astrocytes [15]. Complex intracellular pathways in the neuron and astrocytes lead to release of Nitric oxide (NO), EET and PGE_2_ that act on the vascular smooth muscle or pericytes, leading to alteration in vessel diameter thus regulating the blood flow [16–18].

Understanding the NV unit and coupling opened up gateways to explore the dynamic interactions underlying the normal physiological processes like memory, cerebral auto-regulation during sensory stimulus and motor task, and also in imaging techniques like blood oxygen level dependent signal(BOLD) fMRI [19]. This review focuses on the cerebral and retinal NV coupling in relation to AD. The detailed analysis regarding NV unit in brain function and disease is described elsewhere [20].

### Cerebral neurovascular coupling in AD

In the late 1980’s a series of studies were published hypothesizing the role of blood flow dysregulation and cerebral hypoperfusion in AD [20,21]. Cerebral blood flow was significantly lower in the bilateral temporal lobes of AD patients compared to age matched controls. There was also a global cerebral blood flow reduction in more severe cases [22]. These observations were further supported by the findings of Montardi [23] and Eberling[24].

### Dissecting the pathology at the three levels of NV unit

#### Synaptic excitability

Cholinergic innervation is the drive for neuronal excitability in the hippocampal region which is lost in AD due to degeneration of Nucleus basalis of Meynert (NBM) [25]. Electrical excitation of NBM results in an increase in regional cerebral blood flow in the hippocampal region Vascular dysregulation in the hippocampal region of AD patients depresses glucose metabolism by down regulating the GLUT1 receptors of the vascular endothelial cells leading to neuronal damage [26]. This emphasizes the role of the synapse in blood flow regulation.

#### Vascular pathology

Thickening of basement membrane and accumulation of amyloid beta in the capillaries are observed in the mouse models of AD. In addition, smooth muscle cells involved in vascular contractility also show abnormal constrictions and degeneration [27,28].

#### Glia

Attwell et al. in 2010 [11] showed swelling and retraction of the astrocytic foot processes in the mouse model of AD.

With this complex pathobiology of AD, studying, analyzing and quantifying these dynamic changes at three distinct and integrated structures are critical tasks. Further diagnosing the disease based on these parameters remains a major hurdle as the diagnostic tool should have moderate sensitivity, specificity and preferably be noninvasive.

### Diagnostic tools

The available diagnostic tools are Single Photon Emission Computer Tomography (SPECT), Transcranial Doppler (TCD) and Near Infrared Spectroscopy (NIRS). SPECT is the commonly used technique to assess regional cerebral blood flow [29]; but it has its own limitations. All previous studies so far discussed used SPECT which uses radioactive isotopes. TCD was first described by Aaslid and it evaluates the neurovascular coupling in humans in both health and disease [9,10]. It can also measure cerebral blood flow velocity in the main intracranial vessels non-invasively and with high accuracy. The findings in main blood vessels are reciprocated to the auto-regulatory mechanisms at the level of microvasculature which are physiologically different. The advantages of TCD are low cost, ease of use and good temporal resolution. NIRS technique helps to indirectly quantify the oxygenated and deoxygenated hemoglobin levels based on the relative transparency of the tissue to near infrared rays. Thus, it determines the cerebral autoregulatory mechanisms in sensory, motor and cognitive tasks. The results of TCD and NIRS techniques complement each other. TCD provides information on blood flow whereas NIRS provides information on the neuronal viability. Ideally these complementary inferences should be congruent to each other. But practically, only minimal congruence is noticed between them. The results quantified using semi-automated softwares introduced by Phillips et al. [8] can provide both temporal and amplitudanal relationship between neuronal excitability and cerebral auto-regulation. This could be considered as a preliminary step in the long journey towards the quantitative analysis of dynamic cerebral autoregulatory mechanisms.

### Retinal NV coupling

Retina, an embryological derivative of the central nervous system (CNS), is a layered structure considered to be anatomical and physiological simulant of the CNS [30]. With this, it is inferred that the pathophysiological processes in the brain are reflected in the retina. In addition, retina is an easily accessible tissue hence it is explored in study of degenerative brain disorders [31,32]. NV unit of retina consists of the retinal ganglion cells, astrocytes, Muller cells (which are the retinal glial cells), microvasculature (branches of the central retinal artery from the ophthalmic artery) and the pericytes [33]. Dynamic NV coupling is also observed in retina similar to brain. Retinal blood flow is modulated by glial and synaptic interactions. The role of retinal glial cells in retinal vascular autoregulation is highly appreciated [34].

### Retinal NV coupling in AD

Having an understanding about the physiological phenomenon of NV coupling in the retina and brain, it can be speculated that retina can mirror the dysregulatory mechanisms observed in NV unit of brain.

### Dissecting the pathology at three levels of retinal NV unit

#### Inner retina

Structural changes are evident in the inner plexiform layer (IPL), ganglion cell layer (GCL) and retinal nerve fiber layer (RNFL) of patients with AD and Mild Cognitive Impairment (MCI) [35]. However, the layer that is initially affected remains unknown. Significant reduction of the ganglion cell neuronal density is observed in the temporal region at eccentricities of the central retina (0.0–0.5 mm, 0.5–1.0 mm, 1.0–1.5 mm; 52%, 38% and 38%) of the AD patients. Number of ganglion cell neurons was reduced to 38.7% in the peripheral retina of AD patients compared to the control subjects [36,37]. Thinning of Ganglion cell-inner plexiform layer (GC-IPL) complex in all six quadrants (superior, superonasal, inferonasal, inferior, inferotemporal, and superotemporal) has been shown in AD patients. It has also been showed that MCI patients have significant reduction in GC-IPL thicknesses [38]. Structural changes in the IPL are also observed in the transgenic mouse model of AD. There is a significant reduction (34.4%) in the ganglion cell dentritic complexity in these AD mice compared to the wild type [39].

#### Retinal Nerve Fiber Layer (RNFL)

RNFL thinning was also demonstrated in few studies. Among a group of nine AD cases and eight age matched controls, significant thinning of the RNFL mainly in the superior quadrant was shown in AD patients [40]. RNFL layer thickness was measured in several clock hour positions in a cohort of 25 AD patients, 26 MCI patients and 21 controls. It showed significant RNFL thinning specifically in 5 and 6 clock hour positions (inferior quadrant) in the MCI group compared with AD group. MCI group had significant thinning in the temporal quadrant (8, 9, 10 o ‘clock positions) when compared to control group [41]. Typical double peak manner of the RNFL curve was also lost in AD patients [42]. Similar observations were made in the mild, moderate and severe AD patients [43]. These observations reflect the cerebral pathophysiology such as reduction in peripapillary RNFL thickness and macular GCC thickness with a significant increase in the global loss volume (GLV) rate in AD patients. Further, patients having thinner RNFL in the inferior quadrant had a greater chance of converting into AD with severe cognitive disturbances explaining the prognostic value of the RNFL assessment [44].

#### Blood vessel

Abnormalities of central retinal vessels and choroidal circulation are appreciated in AD pathophysiology. Venous diameter, blood velocity and blood flow differed significantly in AD cases [40]. Further using semi-automated software, Singapore1, vessel Assessment (S1VA) it was shown that venular caliber was narrowed, arteriolar and venular fractal dimensions were smaller, and arteriolar and venular tortuosity were higher in AD patients [45]. Persons with low cognitive testing scores (Delayed word recall-DWR, Digit symbol subtest-DST, Word fluency test-WT) were prone to have retinopathy as evidenced by microaneurysms, retinal hemorrhages, soft exudates, hard exudates, macular edema, intraretinal microvascular abnormalities, venous beading, neovascularization [46]. Subsequently, retinopathy prognosticates cognitive decline [47].

Choroid vasculature which is the main source of nourishment to the outer retina, including photoreceptor cells and Retinal Pigment Epithelium (RPE) was also abnormal in AD. Using Spectral Domain Optical Coherence Tomogram (SD OCT) and the Enhanced Depth Imaging (EDI) technique, subfoveal, temporal, nasal, superior, and inferior choroidal thinning was shown in mild to moderate AD patients [48]. This thinning also correlates with the cognitive scores [49].

### Tools for Analyzing the Retinal NV Unit Components

Optical Coherence tomogram (OCT) is used to study retina. Spectral domain OCT, analyses individual layers with great precision. The doppler technology as an add-on to the Spectral OCT can be used to visualize changes in blood flow in the retinal circulation [50]. Enhanced Depth Imaging (EDI) OCT helps to visualize and analyze structures in the outer retina. Oxygen saturation of hemoglobin can be measured using imaging with spectrophotometric noninvasive retinal oximeter. Semi-automated software (S1VA) and Lie analysis [51] for analyzing the vasculature of the retina provide objective estimation of the burden of microvascular disease in the eye. Complexities in the geometrics of microvasculature are dealt with using brain inspired multi-orientation approach [51]. The same tool is hypothesized to be a game changer in analyzing the retinal images in neurodegenerative disorders. Semi-automated softwares (calorimetric analysis software: Laguna ONhe) are also used in analyzing the optic disc color changes which are noted in neurodegenerative disorders like AD [52]. Thus, automated software and mathematical algorithms are used for vessel tracking, estimation of topographical parameters like vessel tortuosity, branching angle, junctional exponent and fractal properties.

### Controverting Evidences

Having briefly described the existing literature on the individual components of the NV unit, we now highlight the lacunae in the knowledge of NV unit as a whole in AD and stress the importance of analyzing the unit holistically through studies contradicting the above results. It is shown that in a cohort of 21 AD patients and 21 controls, the mean peri-papillary RNFL thickness in all 4 quadrants were similar in AD and control subjects. The central subfield retinal thickness in the two groups did not differ either [48], contrasting the afore mentioned studies. Also, it is shown that there is no correlation between cognitive status (MMSE scores) and OCT parameters in AD patients [41]. The quadrant of the retina which shows RNFL thinning is highly variable in different studies [36,37]. Animal models of AD showed no change in the retinal ganglion cell number and retinal vasculature [53], which is in contradiction to the human autopsy study [37]. We speculate that these contradicting results in various studies point to the lack of integrated analysis of the NV unit as a whole and the lacunae in understanding the role of glia in NV coupling.

## Discussion

The aim of this review article is to highlight the need for evaluating the NV unit as a whole rather than the individual changes at the level of individual structures within the unit, like the nerve or the blood vessel. Analyzing this complex structure in real time is indeed a tedious exercise. Retinal changes are not only specific to neurodegenerative diseases, they are also evident in eye diseases such as glaucoma, and age related macular degeneration, systemic diseases and cardiovascular disorders. The fact is that all these studies have considered a single structure in the complex NV unit and tried to correlate with the cognitive changes. None of these studies had combined vascular parameters with the neuro-glial interactions. Though neuro-glial interaction is itself a complex phenomenon in its own, it can still be studied. Combining all the parameters would be highly beneficial. This multi-parametric analysis will aid us in gaining more insights into the pathophysiology of the disease process in the retina and also will overcome the major hurdle of low sensitivity and specificity. Thus this article proposes the merging of different parameters; RNFL with any of the topographical parameters estimated objectively using automated softwares, can significantly differentiate patients with AD from control group. Later, using high end OCT, quantification of oxygen saturation in human retinal vasculature can be attempted. The geometry of individual cell scan is studied using Ultra High Resolution-OCT and the specific protein or chemical composition can be determined using Molecular imaging OCT.

## Conclusion

Thus, this review can serve as a bird’s eye view on the afore discussed problems and limitations associated with the individual parameter assessment and it also emphasizes a tangible solution of combining different approaches in a holistic way.
